# Supplementary material for: Heparin-based hydrogel scaffolding alters the transcriptomic profile and increases the chemoresistance of MDA-MB-231 triple-negative breast cancer cells
Source: Biomater Sci. 2020 Feb 13;8(10):2786–96. doi: 10.1039/c9bm01481k (PMC7497406; doi:10.1039/c9bm01481k)
Supplement: Supplementary file 2 [file BM-008-C9BM01481K-s002.zip › Supplementary File 4/HP-B hydrogel + EGF (s)vControl/Pathways/my_analysis.Gsea.1545201170987/HALLMARK_EPITHELIAL_MESENCHYMAL_TRANSITION.html]

Details for gene set HALLMARK\_EPITHELIAL\_MESENCHYMAL\_TRANSITION[GSEA]

|  || Dataset | expr.class.cls#HYDEGF\_versus\_CONTROL.class.cls#HYDEGF\_versus\_CONTROL\_repos |
| Phenotype | class.cls#HYDEGF\_versus\_CONTROL\_repos |
| Upregulated in class | CONTROL |
| GeneSet | HALLMARK\_EPITHELIAL\_MESENCHYMAL\_TRANSITION |
| Enrichment Score (ES) | -0.42014992 |
| Normalized Enrichment Score (NES) | -1.949935 |
| Nominal p-value | 0.0 |
| FDR q-value | 2.926038E-4 |
| FWER p-Value | 0.002 |
Table: GSEA Results Summary

  

Fig 1: Enrichment plot: HALLMARK\_EPITHELIAL\_MESENCHYMAL\_TRANSITION      
 Profile of the Running ES Score & Positions of GeneSet Members on the Rank Ordered List

  

| PROBE | DESCRIPTION (from dataset) | GENE SYMBOL | GENE\_TITLE | RANK IN GENE LIST | RANK METRIC SCORE | RUNNING ES | CORE ENRICHMENT || 1 | EFEMP2 | na |  |  | 247 | 2.477 | 0.0034 | No |
| 2 | GADD45B | na |  |  | 524 | 2.096 | 0.0028 | No |
| 3 | FBLN1 | na |  |  | 689 | 1.914 | 0.0068 | No |
| 4 | MYLK | na |  |  | 828 | 1.802 | 0.0115 | No |
| 5 | COL6A3 | na |  |  | 1059 | 1.646 | 0.0103 | No |
| 6 | TNFRSF12A | na |  |  | 1130 | 1.611 | 0.0173 | No |
| 7 | PVR | na |  |  | 1204 | 1.566 | 0.0238 | No |
| 8 | MCM7 | na |  |  | 1322 | 1.505 | 0.0276 | No |
| 9 | FSTL3 | na |  |  | 1328 | 1.503 | 0.0373 | No |
| 10 | ACTA2 | na |  |  | 1336 | 1.499 | 0.0469 | No |
| 11 | ID2 | na |  |  | 1377 | 1.479 | 0.0545 | No |
| 12 | OXTR | na |  |  | 1826 | 1.318 | 0.0397 | No |
| 13 | COL1A1 | na |  |  | 1873 | 1.303 | 0.0459 | No |
| 14 | ECM2 | na |  |  | 1886 | 1.299 | 0.0539 | No |
| 15 | PMEPA1 | na |  |  | 1964 | 1.277 | 0.0583 | No |
| 16 | COL4A2 | na |  |  | 1989 | 1.269 | 0.0654 | No |
| 17 | COL16A1 | na |  |  | 2059 | 1.249 | 0.0701 | No |
| 18 | VEGFC | na |  |  | 2234 | 1.201 | 0.0689 | No |
| 19 | COL5A2 | na |  |  | 2434 | 1.144 | 0.0660 | No |
| 20 | GADD45A | na |  |  | 2798 | 1.052 | 0.0539 | No |
| 21 | COL7A1 | na |  |  | 2849 | 1.041 | 0.0582 | No |
| 22 | TAGLN | na |  |  | 2945 | 1.017 | 0.0599 | No |
| 23 | APLP1 | na |  |  | 3214 | 0.956 | 0.0522 | No |
| 24 | ADAM12 | na |  |  | 3301 | 0.943 | 0.0539 | No |
| 25 | COL8A2 | na |  |  | 3353 | 0.933 | 0.0574 | No |
| 26 | ABI3BP | na |  |  | 3416 | 0.919 | 0.0602 | No |
| 27 | TPM2 | na |  |  | 3484 | 0.908 | 0.0627 | No |
| 28 | CTGF | na |  |  | 3508 | 0.903 | 0.0675 | No |
| 29 | LAMA2 | na |  |  | 3609 | 0.883 | 0.0680 | No |
| 30 | GPC1 | na |  |  | 3618 | 0.882 | 0.0735 | No |
| 31 | COL12A1 | na |  |  | 4059 | 0.802 | 0.0557 | No |
| 32 | LOXL1 | na |  |  | 4517 | 0.732 | 0.0365 | No |
| 33 | COL5A1 | na |  |  | 4746 | 0.696 | 0.0292 | No |
| 34 | CD44 | na |  |  | 5293 | 0.607 | 0.0045 | No |
| 35 | BDNF | na |  |  | 5346 | 0.597 | 0.0057 | No |
| 36 | JUN | na |  |  | 5642 | 0.555 | -0.0061 | No |
| 37 | SNTB1 | na |  |  | 5662 | 0.552 | -0.0034 | No |
| 38 | COL1A2 | na |  |  | 5782 | 0.534 | -0.0062 | No |
| 39 | CAP2 | na |  |  | 5864 | 0.523 | -0.0069 | No |
| 40 | FBN1 | na |  |  | 6535 | 0.433 | -0.0393 | No |
| 41 | NOTCH2 | na |  |  | 7202 | 0.343 | -0.0719 | No |
| 42 | PDGFRB | na |  |  | 7303 | 0.329 | -0.0750 | No |
| 43 | TGFBR3 | na |  |  | 7326 | 0.326 | -0.0740 | No |
| 44 | CADM1 | na |  |  | 7418 | 0.314 | -0.0767 | No |
| 45 | VCAN | na |  |  | 7533 | 0.301 | -0.0807 | No |
| 46 | DKK1 | na |  |  | 7601 | 0.292 | -0.0823 | No |
| 47 | PLOD3 | na |  |  | 7602 | 0.292 | -0.0804 | No |
| 48 | TPM1 | na |  |  | 7718 | 0.276 | -0.0846 | No |
| 49 | FSTL1 | na |  |  | 8207 | 0.222 | -0.1087 | No |
| 50 | BASP1 | na |  |  | 8228 | 0.218 | -0.1083 | No |
| 51 | MYL9 | na |  |  | 8322 | 0.207 | -0.1119 | No |
| 52 | TNFRSF11B | na |  |  | 8824 | 0.144 | -0.1372 | No |
| 53 | HTRA1 | na |  |  | 8915 | 0.133 | -0.1410 | No |
| 54 | LRRC15 | na |  |  | 9121 | 0.111 | -0.1511 | No |
| 55 | QSOX1 | na |  |  | 9271 | 0.093 | -0.1583 | No |
| 56 | BMP1 | na |  |  | 9326 | 0.085 | -0.1606 | No |
| 57 | MATN3 | na |  |  | 9620 | 0.058 | -0.1756 | No |
| 58 | COL4A1 | na |  |  | 9646 | 0.056 | -0.1765 | No |
| 59 | NID2 | na |  |  | 9949 | 0.024 | -0.1922 | No |
| 60 | PTHLH | na |  |  | 9984 | 0.024 | -0.1938 | No |
| 61 | SNAI2 | na |  |  | 10596 | -0.050 | -0.2256 | No |
| 62 | PCOLCE2 | na |  |  | 10786 | -0.077 | -0.2350 | No |
| 63 | ITGB5 | na |  |  | 11075 | -0.118 | -0.2493 | No |
| 64 | LOXL2 | na |  |  | 11483 | -0.161 | -0.2696 | No |
| 65 | MEST | na |  |  | 11757 | -0.197 | -0.2826 | No |
| 66 | CALD1 | na |  |  | 11772 | -0.199 | -0.2821 | No |
| 67 | RHOB | na |  |  | 11865 | -0.214 | -0.2855 | No |
| 68 | IL15 | na |  |  | 12671 | -0.309 | -0.3257 | No |
| 69 | MAGEE1 | na |  |  | 12692 | -0.312 | -0.3247 | No |
| 70 | FZD8 | na |  |  | 12693 | -0.312 | -0.3226 | No |
| 71 | PDLIM4 | na |  |  | 12694 | -0.313 | -0.3205 | No |
| 72 | ELN | na |  |  | 12943 | -0.335 | -0.3313 | No |
| 73 | CAPG | na |  |  | 12979 | -0.337 | -0.3310 | No |
| 74 | TNC | na |  |  | 13218 | -0.374 | -0.3410 | No |
| 75 | LRP1 | na |  |  | 13236 | -0.376 | -0.3394 | No |
| 76 | VCAM1 | na |  |  | 13271 | -0.382 | -0.3386 | No |
| 77 | SDC1 | na |  |  | 13322 | -0.390 | -0.3387 | No |
| 78 | LAMA3 | na |  |  | 13513 | -0.415 | -0.3459 | No |
| 79 | SPOCK1 | na |  |  | 13532 | -0.418 | -0.3441 | No |
| 80 | FAS | na |  |  | 13787 | -0.456 | -0.3544 | No |
| 81 | THBS2 | na |  |  | 13821 | -0.462 | -0.3531 | No |
| 82 | PCOLCE | na |  |  | 13967 | -0.483 | -0.3575 | No |
| 83 | SLC6A8 | na |  |  | 14163 | -0.502 | -0.3644 | No |
| 84 | PLAUR | na |  |  | 14166 | -0.503 | -0.3612 | No |
| 85 | MMP3 | na |  |  | 14191 | -0.504 | -0.3591 | No |
| 86 | TGFB1 | na |  |  | 14260 | -0.515 | -0.3593 | No |
| 87 | FERMT2 | na |  |  | 14467 | -0.551 | -0.3665 | No |
| 88 | LAMC1 | na |  |  | 14471 | -0.552 | -0.3630 | No |
| 89 | COL6A2 | na |  |  | 14661 | -0.583 | -0.3691 | No |
| 90 | INHBA | na |  |  | 14670 | -0.585 | -0.3656 | No |
| 91 | ENO2 | na |  |  | 14703 | -0.591 | -0.3634 | No |
| 92 | RGS4 | na |  |  | 15008 | -0.634 | -0.3751 | No |
| 93 | SERPINH1 | na |  |  | 15083 | -0.648 | -0.3747 | No |
| 94 | WIPF1 | na |  |  | 15234 | -0.675 | -0.3782 | No |
| 95 | GPX7 | na |  |  | 15382 | -0.700 | -0.3812 | No |
| 96 | PLOD1 | na |  |  | 15757 | -0.775 | -0.3957 | No |
| 97 | PFN2 | na |  |  | 15776 | -0.779 | -0.3915 | No |
| 98 | VIM | na |  |  | 16205 | -0.871 | -0.4082 | No |
| 99 | EMP3 | na |  |  | 16303 | -0.898 | -0.4074 | No |
| 100 | COPA | na |  |  | 16547 | -0.956 | -0.4138 | Yes |
| 101 | GJA1 | na |  |  | 16597 | -0.968 | -0.4100 | Yes |
| 102 | CXCL1 | na |  |  | 16603 | -0.969 | -0.4038 | Yes |
| 103 | TIMP1 | na |  |  | 16662 | -0.990 | -0.4003 | Yes |
| 104 | TGFBI | na |  |  | 16713 | -1.002 | -0.3963 | Yes |
| 105 | TGM2 | na |  |  | 16812 | -1.030 | -0.3947 | Yes |
| 106 | DPYSL3 | na |  |  | 16829 | -1.037 | -0.3887 | Yes |
| 107 | TNFAIP3 | na |  |  | 16896 | -1.053 | -0.3852 | Yes |
| 108 | CTHRC1 | na |  |  | 16991 | -1.088 | -0.3829 | Yes |
| 109 | GLIPR1 | na |  |  | 17107 | -1.114 | -0.3816 | Yes |
| 110 | ITGA5 | na |  |  | 17223 | -1.152 | -0.3800 | Yes |
| 111 | MATN2 | na |  |  | 17264 | -1.166 | -0.3744 | Yes |
| 112 | FUCA1 | na |  |  | 17274 | -1.170 | -0.3671 | Yes |
| 113 | PPIB | na |  |  | 17331 | -1.194 | -0.3622 | Yes |
| 114 | LOX | na |  |  | 17366 | -1.203 | -0.3560 | Yes |
| 115 | NT5E | na |  |  | 17372 | -1.205 | -0.3483 | Yes |
| 116 | ECM1 | na |  |  | 17433 | -1.227 | -0.3433 | Yes |
| 117 | GEM | na |  |  | 17445 | -1.234 | -0.3357 | Yes |
| 118 | PLOD2 | na |  |  | 17461 | -1.240 | -0.3283 | Yes |
| 119 | SLIT2 | na |  |  | 17519 | -1.266 | -0.3229 | Yes |
| 120 | SERPINE2 | na |  |  | 17637 | -1.308 | -0.3204 | Yes |
| 121 | IL32 | na |  |  | 17743 | -1.353 | -0.3170 | Yes |
| 122 | SFRP1 | na |  |  | 17787 | -1.375 | -0.3102 | Yes |
| 123 | LGALS1 | na |  |  | 17822 | -1.398 | -0.3027 | Yes |
| 124 | PTX3 | na |  |  | 17871 | -1.426 | -0.2958 | Yes |
| 125 | DAB2 | na |  |  | 17915 | -1.448 | -0.2885 | Yes |
| 126 | MMP1 | na |  |  | 17947 | -1.463 | -0.2804 | Yes |
| 127 | PMP22 | na |  |  | 18110 | -1.553 | -0.2786 | Yes |
| 128 | WNT5A | na |  |  | 18187 | -1.597 | -0.2721 | Yes |
| 129 | IL6 | na |  |  | 18227 | -1.619 | -0.2634 | Yes |
| 130 | TPM4 | na |  |  | 18344 | -1.707 | -0.2582 | Yes |
| 131 | CALU | na |  |  | 18483 | -1.826 | -0.2534 | Yes |
| 132 | SGCB | na |  |  | 18513 | -1.847 | -0.2427 | Yes |
| 133 | SPARC | na |  |  | 18543 | -1.861 | -0.2319 | Yes |
| 134 | IGFBP3 | na |  |  | 18665 | -2.008 | -0.2250 | Yes |
| 135 | TIMP3 | na |  |  | 18706 | -2.053 | -0.2135 | Yes |
| 136 | SDC4 | na |  |  | 18719 | -2.074 | -0.2004 | Yes |
| 137 | ITGAV | na |  |  | 18729 | -2.091 | -0.1871 | Yes |
| 138 | PRSS2 | na |  |  | 18762 | -2.129 | -0.1747 | Yes |
| 139 | LAMC2 | na |  |  | 18772 | -2.148 | -0.1609 | Yes |
| 140 | NNMT | na |  |  | 18828 | -2.244 | -0.1490 | Yes |
| 141 | SCG2 | na |  |  | 18839 | -2.267 | -0.1345 | Yes |
| 142 | AREG | na |  |  | 18856 | -2.288 | -0.1202 | Yes |
| 143 | CD59 | na |  |  | 18886 | -2.333 | -0.1063 | Yes |
| 144 | CDH11 | na |  |  | 18932 | -2.418 | -0.0927 | Yes |
| 145 | EDIL3 | na |  |  | 18945 | -2.445 | -0.0772 | Yes |
| 146 | ITGA2 | na |  |  | 18964 | -2.492 | -0.0616 | Yes |
| 147 | ITGB3 | na |  |  | 18981 | -2.535 | -0.0457 | Yes |
| 148 | TFPI2 | na |  |  | 18994 | -2.568 | -0.0293 | Yes |
| 149 | PRRX1 | na |  |  | 19101 | -2.909 | -0.0157 | Yes |
| 150 | SAT1 | na |  |  | 19134 | -3.131 | 0.0034 | Yes |
Table: GSEA details [plain text format]

  

Fig 2: HALLMARK\_EPITHELIAL\_MESENCHYMAL\_TRANSITION      
 Blue-Pink O' Gram in the Space of the Analyzed GeneSet

  

Fig 3: HALLMARK\_EPITHELIAL\_MESENCHYMAL\_TRANSITION: Random ES distribution      
 Gene set null distribution of ES for **HALLMARK\_EPITHELIAL\_MESENCHYMAL\_TRANSITION**
